# Deep characterization of the anti-drug antibodies developed in Fabry disease patients, a prospective analysis from the French multicenter cohort FFABRY

**DOI:** 10.1186/s13023-018-0877-4

**Published:** 2018-07-31

**Authors:** Wladimir Mauhin, Olivier Lidove, Damien Amelin, Foudil Lamari, Catherine Caillaud, Federico Mingozzi, Gaëlle Dzangué-Tchoupou, Louiza Arouche-Delaperche, Claire Douillard, Bertrand Dussol, Vanessa Leguy-Seguin, Pauline D’Halluin, Esther Noel, Thierry Zenone, Marie Matignon, François Maillot, Kim-Heang Ly, Gérard Besson, Marjolaine Willems, Fabien Labombarda, Agathe Masseau, Christian Lavigne, Roseline Froissart, Didier Lacombe, Jean Marc Ziza, Eric Hachulla, Olivier Benveniste

**Affiliations:** 1 Sorbonne Université, INSERM, UMR 974, Centre of Research in Myology, Association Institut de Myologie, Pitié-Salpêtrière University Hospital, 75013 Paris, France; 2 Internal Medicine and Rheumatology Department, Diaconesses-Croix Saint Simon Hospital Group, Paris, France; 3 Metabolic Biochemistry Department, Pitié Salpêtrière University Hospital, AP-HP, Paris, France; 4 GRC13-Neurometabolisme- Sorbonne Universités UPMC, Paris 06, Paris, France; 5 Biochemistry, Metabolomic and Proteomic Department, Necker Enfants Malades University Hospital, AP-HP, Paris, France; 6 INSERM U1151, Institute Necker Enfants Malades, Paris Descartes University, Paris, France; 7 Reference Center for Inborn Metabolic diseases, Jeanne de Flandres Hospital, Lille, France; 8 Nephrology Department, Aix Marseille Université et Centre d’Investigation Clinique 1409, INSERM/AMU/AP-HM, Marseille, France; 9 Internal Medicine and Clinical Immunology Department, Francois Mitterrand Hospital, Dijon, France; 10 Nephrology and Clinical Immunology Department, Tours University Hospital, François Rabelais University, Tours, France; 11 Internal Medicine Department, Strasbourg University Hospital, Strasbourg, France; 12 Internal Medicine Department, Valence Hospital, Valence, France; 13 Nephrology and Renal Transplantation Department, Institut Francilien de Recherche en Néphrologie et Transplantation (IFRNT), Henri-Mondor/Albert-Chenevier University Hospital, APHP, Créteil, France; 14 University of Paris-Est-Créteil (UPEC), DHU (Département Hospitalo-Universitaire) VIC (Virus-Immunité-Cancer), IMRB (Institut Mondor de Recherche Biomédicale), Team 21, INSERM U 955, Créteil, France; 15 Internal Medicine Department, Tours University Hospital, University of Tours, UMR INSERM 1253, Tours, France; 16 Internal Medicine Department, Dupuytren University Hospital, Limoges, France; 17 Neurology Department, Grenoble University Hospital, Grenoble, France; 18 Medical Genetics and Rare Diseases Department, Montpellier University Hospital, Montpellier, France; 19 Cardiology Department, Caen University Hospital, Caen, France; 20 Internal Medicine Department, Hôtel-Dieu University Hospital, Nantes, France; 21 Internal Medicine and Vascular Diseases Department, Angers University Hospital, Angers, France; 22 Laboratory for Inborn Errors of Metabolism, East Hospital, Hospices Civils de Lyon, Bron, France; 23 Medical Genetics Department, CHU Bordeaux, INSERM U1211, Bordeaux University, Bordeaux, France; 24 Internal Medicine Department, Huriez Hospital, University of Lille, 59037 Lille, France; 25 Internal Medicine and Clinical Immunology Department, Pitié-Salpêtrière University Hospital, DHU I2B, AP-HP, Paris, France

**Keywords:** Fabry disease, Anti-drug antibodies, Agalsidase, Lysosomal storage disease, Enzyme replacement therapy, IgG4

## Abstract

**Background:**

Fabry disease (OMIM #301500) is an X-linked disorder caused by alpha-galactosidase A deficiency with two major clinical phenotypes: classic and non-classic of different prognosis. From 2001, enzyme replacement therapies (ERT) have been available. We aimed to determine the epidemiology and the functional characteristics of anti-drug antibodies. Patients from the French multicenter cohort FFABRY (*n* = 103 patients, 53 males) were prospectively screened for total anti-agalsidase IgG and IgG subclasses with a home-made enzyme-linked immunosorbent assay (ELISA), enzyme-inhibition assessed with neutralization assays and lysoGb3 plasma levels, and compared for clinical outcomes.

**Results:**

Among the patients exposed to agalsidase, 40% of men (*n* = 18/45) and 8% of women (*n* = 2/25) had antibodies with a complete cross-reactivity towards both ERTs. Antibodies developed preferentially in men with non-missense *GLA* mutations (relative risk 2.88, *p* = 0.006) and classic phenotype (58.6% (17/29) vs 6.7% (1/16), *p* = 0.0005). Specific anti-agalsidase IgG1 were the most frequently observed (16/18 men), but the highest concentrations were observed for IgG4 (median 1.89 μg/ml, interquartile range (IQR) [0.41–12.24]). In the men exposed to agalsidase, inhibition was correlated with the total IgG titer (*r* = 0.67, *p* < 0.0001), especially IgG4 (*r* = 0.75, p = 0.0005) and IgG2 (*r* = 0.72, *p* = 0.001). Inhibition was confirmed intracellularly in Fabry patient leucocytes cultured with IgG-positive versus negative serum (median: 42.0 vs 75.6%, *p* = 0.04), which was correlated with IgG2 (r = 0.67, *p* = 0.017, *n* = 12) and IgG4 levels (*r* = 0.59, *p* = 0.041, *n* = 12). Plasma LysoGb3 levels were correlated with total IgG (*r* = 0.66, *p* = 0.001), IgG2 (r = 0.72, *p* = 0.004), IgG4 (*r* = 0.58, *p* = 0.03) and IgG1 (*r* = 0.55, *p* = 0.04) titers. Within the classic group, no clinical difference was observed but lysoGb3 levels were higher in antibody-positive patients (median 33.2 ng/ml [IQR 20.6–55.6] vs 12.5 [10.1–24.0], *p* = 0.005).

**Conclusion:**

Anti-agalsidase antibodies preferentially develop in the severe classic Fabry phenotype. They are frequently associated with enzyme inhibition and higher lysoGb3 levels. As such, they could be considered as a hallmark of severity associated with the classic phenotype. The distinction of the clinical phenotypes should now be mandatory in studies dealing with Fabry disease and its current and future therapies.

## Background

In Fabry disease (FD, OMIM #301500), mutations in the *GLA* gene (Xq22.1 300,644) lead to a defect in alpha-galactosidase A with a subsequent accumulation of glycosphingolipids, notably globotriaosylceramide (Gb3) and globotriaosylsphingosine (*lyso*Gb3). Two major phenotypes have been distinguished according to the residual enzyme activity. The classic phenotype occurs below 1% of residual activity, with symptoms observed from childhood such as typical acral pain, cornea verticillata or angiokeratomas and prognosis dominated from the third decade by renal insufficiency, cardiac hypertrophy and cerebrovascular involvement [1, 2]. Above 1%, the non-classic late-onset phenotype is characterized by an almost exclusive cardiopathy without pain, ophthalmological or cutaneous lesions [2]. Women can be affected with a mild to severe phenotype depending on the X-inactivation status in each organ [3]. Among the known *GLA* mutations, deletions, frameshifts and nonsense mutations have been clearly associated with the classic phenotype, whereas the phenotype-genotype correlation is less obvious for missense mutations [4]. Currently, enzyme replacement therapy (ERT) with agalsidase alfa (Replagal™, Shire Plc) or agalsidase beta (Fabrazyme®, Genzyme-Sanofi Corp.) is widely accepted to provide benefits in terms of cardiac hypertrophy and renal disease, at least when therapy is initiated in the early stage of the disease [5–7]. However, in some patients, the disease progresses despite ERT [8]. Although neutralizing anti-agalsidase antibodies have been identified, few studies have investigated the clinical impact of these antibodies [9]. We aimed to determine the significance of anti-agalsidase antibodies and their effects on enzyme activity and the associated clinical manifestations.

## Methods

### Patients and blood samples

The multicenter cohort FFABRY prospectively gathers clinical data and biological samples from patients with an enzymatic and/or genetic diagnosis of FD. The patients were sorted according to their phenotype: patients with a missense mutation and the absence of acral pain or cornea verticillata were referred to as *non-classic* Fabry patients, others were referred to as *classic*. Legal authorizations were obtained from the *Comité consultatif sur le traitement de l’information en matière de recherche dans le domaine de la santé (n°14.324bis*) according to the relevant French legislation. Clinical data were prospectively collected through a standardized online form. Blood samples were collected at the time of inclusion. The samples were centralized in our research unit for isolation of peripheral blood mononuclear cells (PBMCs) using Ficoll-Hypaque™ gradient centrifugation technique before congelation at − 80 °C in fetal-calf-serum (Life Technologies, Saint-Aubin, France, Catalog # 10270106) supplemented with 10% Dimethyl Sulfoxide at − 80 °C. Serum and plasma were isolated by centrifugation using BD Vacutainer™ serum tubes with increased silica act clot activator and BD Vacutainer™ heparin tubes respectively, before congelation at − 80 °C.

### Enzyme-linked immunosorbent assay (ELISA)

ELISA plates (96-well Nunc® Maxisorp, Denmark) were coated with 5 μg/ml of agalsidase alfa (Replagal™, Shire) or beta (Fabrazyme®, Sanofi-Genzyme) or with intravenous immunoglobulin (Clairyg®, LFB Biomédicaments Corp) as a control. The intrinsic background of each serum sample was controlled with *uncoated* wells filled with Dulbecco’s phosphate-buffered saline (DPBS). The plates were blocked with 2% DPBS-bovine serum albumin (DPBS-BSA) and filled with either patient sera (1:100 and subsequent serial two-fold dilutions if positive) or a polyclonal rabbit anti-alpha-galactosidase A antibody (Proteintech®, Manchester, UK) as a control. Goat anti-human IgG (1:20,000 dilution, Novex®, Thermo Scientific™, France) or goat anti-rabbit IgG (1:10,000 dilution, Jackson ImmunoResearch Lab®, USA) both coupled to horseradish peroxidase were used as secondary antibodies for the positive control wells. After incubation with tetramethyl benzidine (TMB, Biolegend®), the reaction was stopped (1 M H_3_PO4), and the absorbance was measured with the Spark 10 M® reader (Tecan Trading AG, Switzerland). Thresholds were determined as an absorbance > the mean + 3 SD of the results from sera obtained from 83 healthy subjects.

For the IgG1–4 subclasses, protocols were adapted using specific secondary monoclonal mouse anti-human IgG1, IgG2, IgG3 and IgG4 biotin-conjugated antibodies (Sigma-Aldrich) and HRP-streptavidin (Biolegend®, USA).

### Neutralizing assay in serum

Microplate wells (optiplate-96 black, Perkin Elmer®) were filled with 30 μl of 4% BSA-H_2_O, 10 μl of agalsidase (2.5 ng/μl) and 10 μl of patient sera or 10 μl of additional 4% BSA-H_2_O. After 10 min incubation, 1 mM 4-methylumbelliferyl-alpha-D-galactopyranoside (Sigma M7633) was added, and fluorescence readings were obtained under kinetic conditions at 37 °C for 1 h in a microplate reader. The residual relative activity (RRA) obtained with sera was defined as the ratio of agalsidase activity measured in sera to the activity measured with only 4% BSA-H_2_O. The basal activities of all sera (without agalsidase) were controlled. All measurements were performed in duplicate. Patients treated with migalastat (Amicus Therapeutics®) were excluded due to the potent enzymatic inhibition by this compound observed in vitro.

### Neutralizing assay in leukocytes

Frozen patient peripheral blood mononuclear cells (PBMCs) were thawed, resuspended and separated into two tubes with agalsidase (5 ng/ml). Fifty microliters of patient sera (related to the patient PBMCs) or 50 μl of fetal bovine serum was added for the RRA determination. The tubes were incubated for 4 h (37 °C, 5% CO_2_). The cells were washed twice in ice-cold phosphate-buffered saline. Viability was assessed with Trypan blue staining before sonication. The protein concentration was determined by bicinchoninic acid (BCA) assay before measuring the enzyme activity. The measurements were performed in duplicate.

### Alpha-N-acetylgalactosaminidase (NAGA) neutralizing assay

Human recombinant NAGA (2.5 and 5 ng/μl, R&D Systems®) was incubated with sodium citrate buffer (pH 4.0) and then patient sera. 4-Nitrophenxyl-N-acetyl-alpxha-D-galactosaminide (2 mM, Sigma-Aldrich®) was added, and the incubation was continued for 10 more min before the addition of NaOH. The absorbance was read at 402 nm. The measurements were performed in duplicate.

### Plasma *lyso*Gb3

The lysoGb3 concentration was measured in available plasma samples by ultra-performance liquid chromatography coupled to tandem mass spectrometry (UPLC-MS/MS). In glass tubes, EDTA-plasma was mixed with glycine-lysoGb3 (100 ng/ml) as an internal standard. Proteins were precipitated with methanol:acetone 1:1 (*v*/v), sonicated and vortexed. After centrifugation, the supernatant was transferred into new tubes and dried. For UPLC-LCMS/MS analysis, the residue was redissolved in methanol. Quantitative analysis of lysoGb3 was performed on a TQD mass spectrometer coupled to an Acquity UPLC system (Waters®) and equipped with an Acquity BEH-C18 column. Elution was achieved by mobile phase A, consisting of 37% methanol, 63% water containing 1 mM ammonium formiate and 0.1% formic acid, and mobile phase B, consisting of 100% methanol containing 1 mM ammonium formiate and 0.1% formic acid. A calibration curve was generated by a serial dilution of lysoGb3 (Matreya-LLC) in methanol, with concentrations ranging from 100 to 1.56 ng/ml. LysoGb3 isoforms were not evaluated.

### Statistical analysis

The estimated glomerular filtration rate (eGFR) according to the Modification of Diet in Renal Disease (MDRD) equation [10] was analyzed using linear regression for the assessment of correlations and analysis of covariance (ANCOVA) for comparisons. The non-parametric Spearman test, Kruskal-Wallis test, Mann-Whitney test and Fisher’s exact t test were used for other variables, such as the concentrations of the interventricular septum thickness, *lyso*Gb3 plasma levels, IgG subclass concentrations and RRA. Logistic regression was used to assess correlations between binary variables and age or time of exposure to agalsidase. Kaplan-Meier analysis with the log-rank test was used for the survival analysis. Missing values were not included in the analyses. GraphPad Prism 5.0 and the EZR plugin version 1.35v [11] packages for the R software were used.

## Results

### Patients

**Fig. 1:**
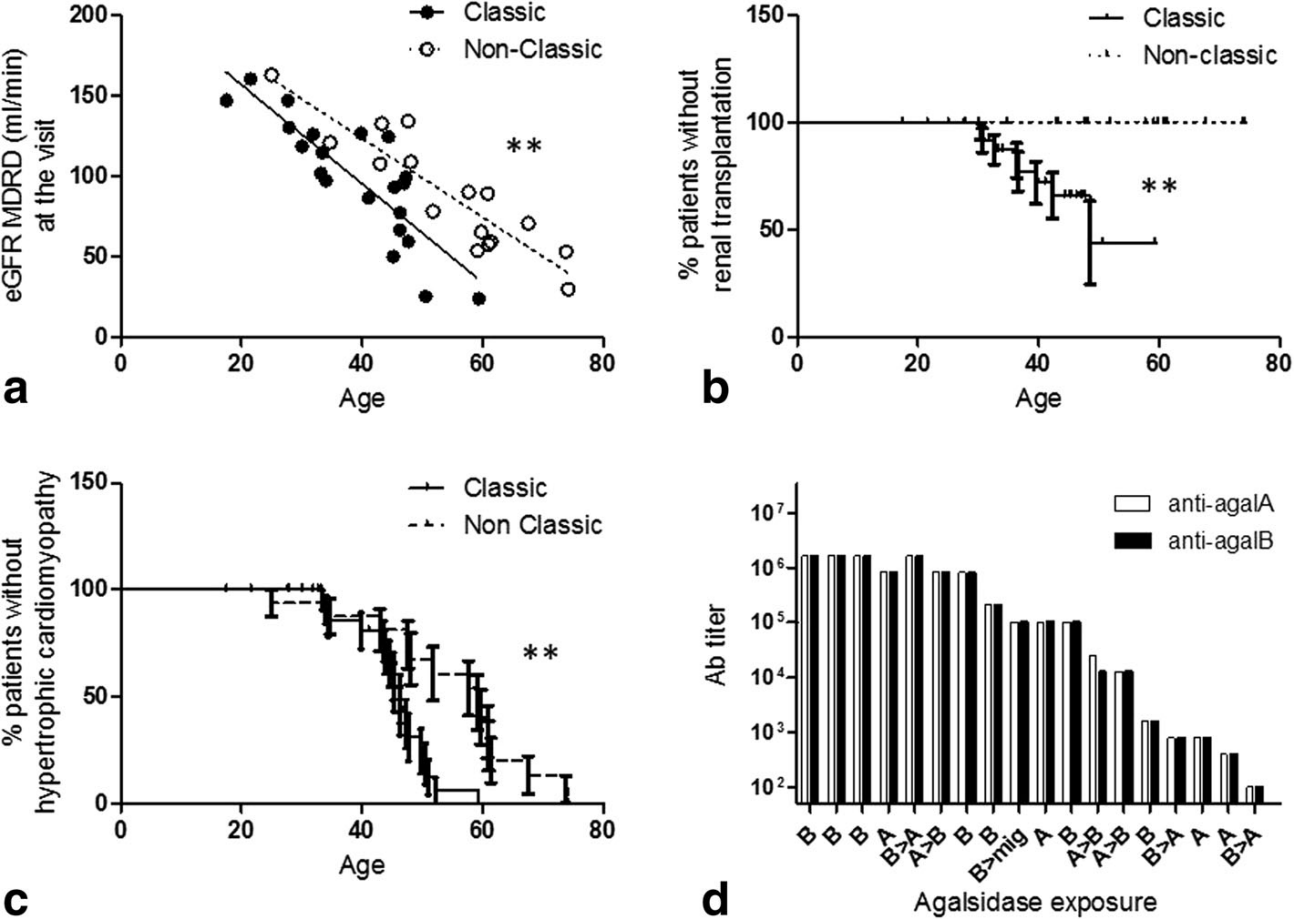
**a** Distribution of the estimated glomerular filtration rates of the treated men according to the classic or non-classic phenotype (linear regression, *p* < 0.001); **b** Risk of renal transplantation according to the phenotype (log-rank test, hazard ratio (HR) classic phenotype = 7.9, *p* = 0.005); **c** Prevalence of hypertrophic cardiomyopathy (HCM; log-rank test, median survival HCM-free 46.3 y in classic patients vs 59.1 y in non-classic patients, HR = 3.96, *p* = 0.02); **d** Antibody titers according to the chronology of the specific treatment received (A: agalsidase alfa; B: agalsidase beta, mig: migalastat). Complete cross reactivity against agalsidase alfa and beta observed for anti-agalsidase antibodies

From December 2014 to January 2017, 103 patients (53 males) with 42 different mutations from 17 different centers were prospectively included in the FFABRY cohort. Among the 50 women, 25 had been exposed to agalsidase (mean age = 52.5 y.; mean cumulated exposure to agalsidase = 6.1 y.), 25 were untreated (mean age = 47.9 y.). Among the men, 8 had not been exposed to agalsidase (mean age 33.2y.) including 5 classic and 3 non-classic patients. Forty-five men had been exposed at least once to ERT, including 29 classic (mean age 40.1 y.; mean cumulated exposure to agalsidase = 8.5 y.) and 16 non-classic Fabry patients (mean age 54.9 y.; mean cumulated exposure = 4.4 y.). As expected, the classic male patients were younger (*p* < 0.001), had longer exposure to agalsidase (*p* < 0.004), worse eGFR evolution (excluding already transplanted patients, ANCOVA, *p* = 0.008, Fig. 1a), higher risk for renal transplantation (log-rank test, hazard ratio (HR) for renal transplantation: 7.9, *p* = 0.005, Fig. 1b) and higher lysoGb3 plasma levels than the non-classic patients (currently treated men only: median 21.1 ng/ml [Interquartile range (IQR) 11.6–37.2] vs 4.5 ng/ml [IQR 2.3–11.3], Mann-Whitney test, *p* = 0.0005). Additionally, hypertrophic cardiomyopathy (HCM) occurred earlier in the classic patients (log rank test, median survival HCM-free 46.3 vs 59.1 y, HR 3.96, *p* = 0.001, Fig. 1c), but the incidence of implantable cardiac devices was not different between the groups (log-rank test, *p* = 0.69).

### Antibodies, genotype and phenotype (Table 1)

Antibodies were prospectively screened in all the patients at the time of inclusion whatever their exposure to agalsidase or their current treatment. In men exposed to agalsidase, 18 (40%) had anti-agalsidase total IgG without any difference in the type of molecules that the men were exposed to either at the time of sampling or previously (alfa 30.8%, beta 44.4%, alfa and beta 42.9%, Kruskal-Wallis test, *p* = 0.73). The cross reactivity was complete (Fig. 1d). Antibody positivity was not dependent on the time of exposure to agalsidase (logistic regression, odds ratio (OR) = 1.1, *p* = 0.09).

**Table 1:** Clinical characteristics of men exposed to agalsidase

	Ab-positive	Ab-negative	n*	*p-*value
N	18	27	45	*–*
Age visit (y.)	43.7 [32.8–49.2]	46.3 [41.4–59.6]	45	*0.12* ^*£*^
Age diag (y.)	28.8 [20.1–41.5]	41.1 [28.1–53.7]	45	*0.09* ^*£*^
Agalsidase exposure A/B/AB	4/8/6	9/11/7	45	*0.70*
Current migalastat	1	1	45	*1* ^*$*^
Cumul. Exp. (y.)	10.6 [3.3–12.2]	4.3 [3.2–7.0]	45	*0.22* ^*£*^
MTP/Missense	10/8	3/21	42^d^	*0.006* ^*$*^
RRA (%)^b^	0.38 [0.25–1.0]	1.10 [0.95–1.1]	43	*0.0003* ^*£*^
LysoGb3^c^ (ng/ml)	25.4 [18.9–48.8]	10.2 [3.1–17.1]	33	*0.0005* ^*£*^
Mainz score total	23.5 [14.0–32.5]	22.0 [15.5–27]	45	*0.74* ^*£*^
Mainz cardiovascular score	2.5 [0.0–9.8]	9.0 [2.5–12.5]	45	*0.14* ^*£*^
Mainz renal score	8.0 [0–18]	0.0 [0.0–8.0]	45	*0.22* ^*£*^
Mainz neurological score	5.5 [2.3–8.8]	5.0 [1.5–8.0]	45	*0.65* ^*£*^
Mainz general score	4.5 [2.5–6.8]	4.0 [1.5–6.5]	45	*0.33* ^*£*^
Dialysis or kidney transplant	6	1	45	*0.012* ^*$*^
Classic/ Non classic phenotype	17/1	12/15	45	*0.0005* ^*$*^

n* number of patients included in the analysis; median [IQR], *RRA*^b^ patients under migalastat were excluded from the analysis, *LysoGb3*^c^ available plasma of patients under agalsidase only, patients under migalastat were excluded from the analysis, *MTPs* mutations leading to a truncated protein (deletion, frameshift or non-sense mutations), *MSSI* Mainz severity score index, *IQR* interquartile range, *RRA* relative residual activity

^d^the genotype was unavailable for 3 patients

^£^Mann-Whitney test; ^$^Fischer’s exact test

Considering phenotypes, antibodies were observed in 58.6% (17/29) of the classic and 6.7% of the non-classic Fabry patients (1/16; Fisher’s exact test, *p* = 0.0005). The non-classic phenotype remained correlated with a lower risk of antibodies when including the time of exposure to agalsidase (logistic regression, Ab positivity OR 0.05, *p* = 0.009). Among the classic patients, there was no difference between the Ab-positive and Ab-negative men concerning the age (Mann-Whitney test, median 43.3 y., [IQR 32.3–48.7] vs 44.4 y. [34.0–46.3] *p* = 0.96) or the time of exposure to agalsidase (Mann-Whitney test, median 11.2 y, [IQR 4.8–13.1] vs 5.9 [4.0–14.1] *p* = 0.81).

**Fig. 2:**
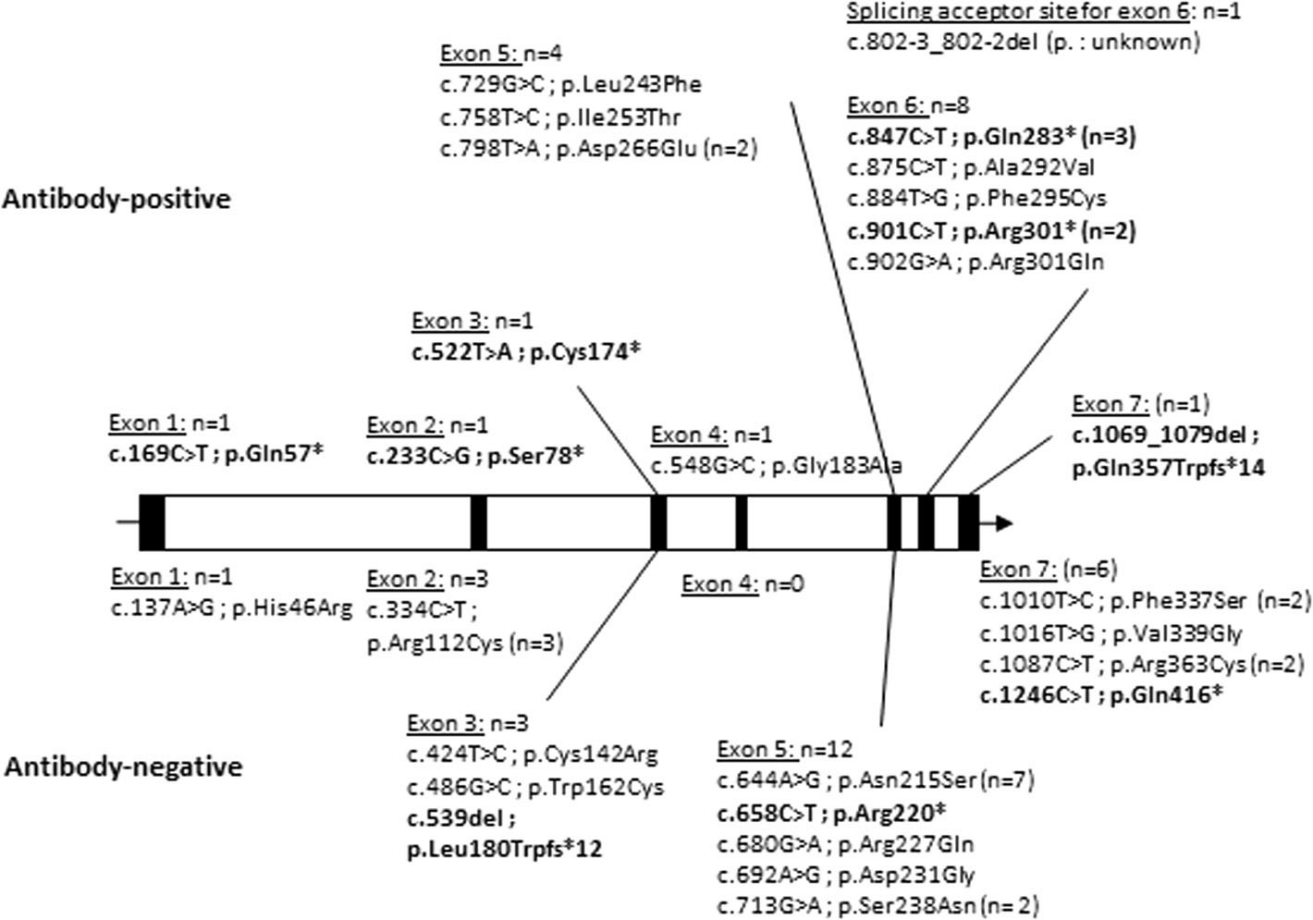
Distribution of GLA mutations observed in men treated with agalsidase; mutations leading to truncated proteins (MTPs) are shown in bold. The genotype was not available for two antibody-negative men

Antibodies were specifically associated with 14 different mutations (Fig. 2, Table 2). Mutations leading to truncated alpha-galactosidase proteins (MTPs), including deletions, nonsense and frameshift mutations (6/13), were more frequently associated with antibodies than missense mutations (55.6% vs 12.0%, *p* = 0.006, Table 2). In the classic men, the association between MTPs and antibodies disappeared (Fisher’s exact test, OR 2.7, *p* = 0.41).

**Table 2:** Characteristics of the antibody-positive patients

	Age (y.)	Mutation		ERT		Ab Titer ag. alfa	Ab Titer ag. beta	IgG1	IgG2	IgG3	IgG4	RRA alfa	RRA beta	*lyso*Gb3 (ng/ml)	Renal event	Clinical phenotype	MSSI				
				Exposure													Total	Cardio-vasc	General	Neuro	Renal
				Type	Time (months)																
1 M	43.8	c.233C>G	p.Ser78*	B	182.0	1,638,400	1,638,400	++++	++++	++++	++++	4%	1%	41.0	yes	Classic	45	15	7	5	18
2 M	33.4	c.847C>T	p.Gln283*	B	159.3	1,638,400	1,638,400	+++	++++	++++	++++	42%	32%	48.0		Classic	14	9	2	3	0
3 M	47.7	c.522T>A	p.Cys174*	B	3.2	1,638,400	1,638,400	++++	+++	+++	+	20%	17%	21.7		Classic	12	0	4	0	8
4 M	46.1	c.901C>T	p.Arg301*	A	78.2	819,200	819,200	++++	++++	++++	++++	41%	35%	60.0		Classic	28	3	11	14	0
5 M	33.2	c.548G>C	p.Gly183Ala	A/B	157.4	1,638,400	1,638,400	++++	++++	+	++++	20%	20%	76.0		Classic	11	0	9	2	0
6 M	43.3	c.169C>T	p.Gln57*	A/B	170.4	819,200	819,200	++++	++++	++++	++++	3%	4%	31.3	yes	Classic	45	17	4	6	18
7 M	47.3	c.847C>T	p.Gln283*	B	156.2	819,200	819,200	++++	++++	++	++++	46%	36%	25.4		Classic	12	10	2	0	0
8 M	50.6	c.802-3_802-2del	unknown	B	16.0	204,800	204,800	++++	++	+++	++++	98%	76%	na		Classic	38	18	5	7	8
9 M	32.7	c.884T>G	p.Phe295Cys	B	46.6	102,400	102,400	++++	no	no	no	mig	mig	10.0	yes	Classic	29	0	2	9	18
10 M	59.1	c.758T>C	p.Ile253Thr	A	7.8	102,400	102,400	++++	no	++	++	75%	61%	10.8		Non classic	23	13	1	1	8
11 M	17.5	c.902G>A	p.Arg301Gln	B	5.1	102,400	102,400	++++	++	+	++++	40%	29%	NA		Classic	14	0	6	8	0
12 M	27.7	c.901C>T	p.Arg301*	A/B	69.3	25,600	12,800	+++	+++	++	++++	54%	38%	48.8		Classic	19	2	6	11	0
13 M	52.1	c.798T>A	p.Asp266Glu	A/B	116.1	12,800	12,800	++++	++	++	no	99%	103%	na	yes	Classic	34	0	8	8	18
14 M	49.8	c.847C>T	p.Gln283*	B	133.5	1600	1600	++++	+	+	no	144%	115%	84.2	yes	Classic	31	7	4	2	18
15 M	41.1	c.1069_1079del	p.Gln357Trpfs*14	A/B	146.3	800	800	no	no	+++	++++	142%	100%	18.9		Classic	24	2	9	5	8
16 M	31.9	c.875C>T	p.Ala292Val	A	135.7	800	800	+++	no	++	++++	114%	93%	15.0		Classic	8	3	2	3	0
17 M	51.0	c.798T>A	p.Asp266Glu	A	120.5	400	400	+	no	no	no	136%	106%	na	yes	Classic	33	0	5	10	18
18 M	27.9	c.729G>C	p.Leu243Phe	A/B	151.0	100	100	no	no	++	no	141%	110%	na		Classic	18	0	4	14	0
19F	65.3	c.695T>C	p.Ile232Thr	A	1.8	6400	6400	no	no	+	no	120%	110%	1.5		NA	19	15	4	0	0
20F	43.2	c.486G>C	p.Trp162Cys	B	10.2	12,800	50	+	no	no	no	126%	113%	4.3		NA	13	0	1	8	4

RRA was considered not informative in the patient #9 under migalastat (mig)

*M* males, *F* female, *A* agalsidase alfa, *B* agalsidase beta, *na* not available, *NA* not applicable; Ab titers observed against agalsidase alfa (ag. alfa) or beta (ag. beta); Renal event defined as estimated glomerular filtration rate below 15 ml/min/1.73m^2^ and/or kidney transplant and/or dialysis; *MSSI* Mainz Severity Score Index with total, cardiovascular (cardio-vasc), general, neurological (neuro) and renal scores

*Non-sense mutations

The IgG subclasses were determined in the 18 Ab-positive men, with samples from 17 IgG-negative treated patients used as controls. All the different IgG subclasses were concomitantly observed in 10/18 cases (Table 2). IgG1 antibodies were the most frequently observed (16/18 men), but the highest concentrations were found for IgG4 (median 1.89 μg/ml, IQR [0.41–12.24]).

Two of the 25 women treated with agalsidase developed anti-agalsidase IgG (8%) 1.8 and 10.8 months after the introduction of agalsidase alfa (titer 1/12,800) and beta (titer 1/6400), respectively. Among the 44/50 available genotypes in the females, both seropositive women carried missense mutations that were not located on exon 6.

Any of the 33 untreated patients had detectable antibodies.

### Biological and clinical outcomes

#### Among the classic men (*n* = 29)

**Fig. 3:**
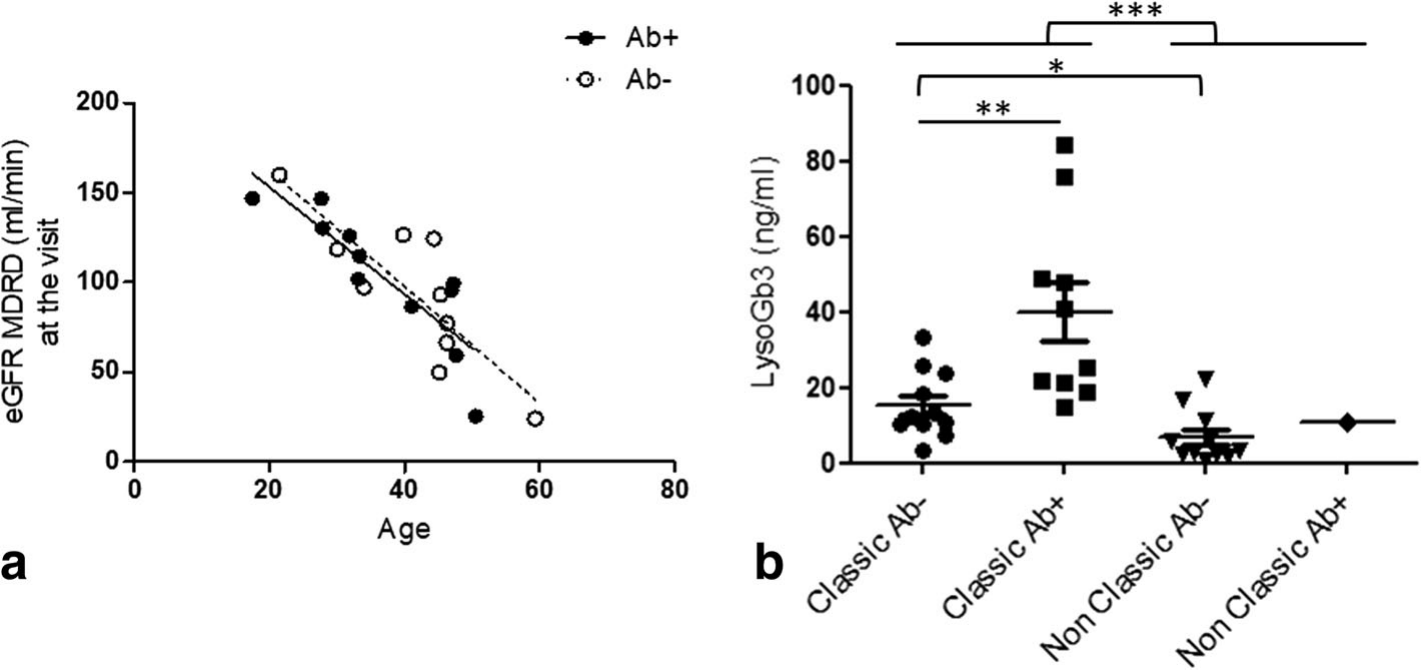
**a** Distribution and linear regression of the estimated glomerular filtration rates of classic patients over the age according to the antibody status; antibody-positive patients: slope − 3.0 ml/min/y, *r*^2^ = 0.75, *p* < 0.001) vs antibody-negative patients (slope − 3.3, *r*^2^ = 0.71, *p* = 0.002; no difference between curves, *p* = 0.79); **b** The lysoGb3 plasma levels in men currently treated with agalsidase according to their phenotype and antibody status. The lysoGb3 levels are higher in classic patients (median 21.1 ng/ml [IQR 11.6–37.2] (*n* = 21) vs 4.5 ng/ml [IQR 2.3–11.3] (*n* = 12), Mann-Whitney test, *p* = 0.0005). This difference is confirmed when considering antibody-negative patients only (median 12.5 ng/ml in classic patients (*n* = 11) vs 3.2 ng/ml in non-classic patients (*n* = 11); *p* = 0.01). Among classic patients only, the lysoGb3 levels are higher in antibody-positive patients (Mann Whitney test, median 33.2 ng/ml [IQR 20.6–55.6] vs 12.5 [10.1–24.0], *p* = 0.005) despite the lack of difference in the time of exposure to agalsidase (Mann Whitney test, median 11.2 y [IQR 4.8–13.1] vs 5.9 [4.0–14.1] *p* = 0.81; data not shown)

The renal outcomes did not differ according to their Ab status. No difference was found in the eGFR slopes between Ab-positive (linear regression, slope − 3.0 ml/min/y, *r*^2^ = 0.75, *p* < 0.001) and Ab-negative non-renal-transplanted patients (linear regression, slope − 3.3, *r*^2^ = 0.71, *p* = 0.002; ANCOVA for comparison, *p* = 0.29, Fig. 3a). No difference was found in the incidence of renal transplantation (log-rank test, *p* = 0.32). Antibody positivity was not correlated with cardiac hypertrophy (logistic regression including age at the visit, *p* = 0.20), stroke (logistic regression including age, *p* = 0.77) or T2-flair-weighted hyperintensities on cerebral magnetic resonance imaging (logistic regression including age, *p* = 0.91). Finally, anti-agalsidase IgGs were not reported to be associated with infusion-related events (anaphylaxis, flu-like syndrome and/or rash; Fisher’s exact test, OR = 4.2, *p* = 0.32).

The lysoGb3 plasma levels were higher in the Ab-positive patients (Mann-Whitney test, median 33.2 ng/ml [IQR 20.6–55.6] vs 12.5 [10.1–24.0], *p* = 0.005, Fig. 3b). LysoGb3 levels were correlated with the total IgG (Spearman’s test, *r* = 0.66, *p* = 0.001), IgG2 (Spearman’s test, *r* = 0.72, *p* = 0.004), IgG4 (*r* = 0.58, *p* = 0.03) and IgG1 (*r* = 0.55, *p* = 0.04) titers.

#### Among the non-classic men (*n* = 16)

Anti-agalsidase antibodies were observed in only one non-classic patient who did not appear as an outlier in the non-classic group in terms of the clinical presentation or lysoGb3 plasma level (10.8 ng/ml in the Ab-positive patient compared to a median level of 3.2 ng/ml [IQR 2.2–11.5] in the Ab-negative patient, Fig. 3b).

#### Among women treated with agalsidase (*n* = 25)

Anti-agalsidase antibodies were observed in two women without any clinical specificity compared to the Ab-negative women.

### Inhibition

**Fig. 4:**
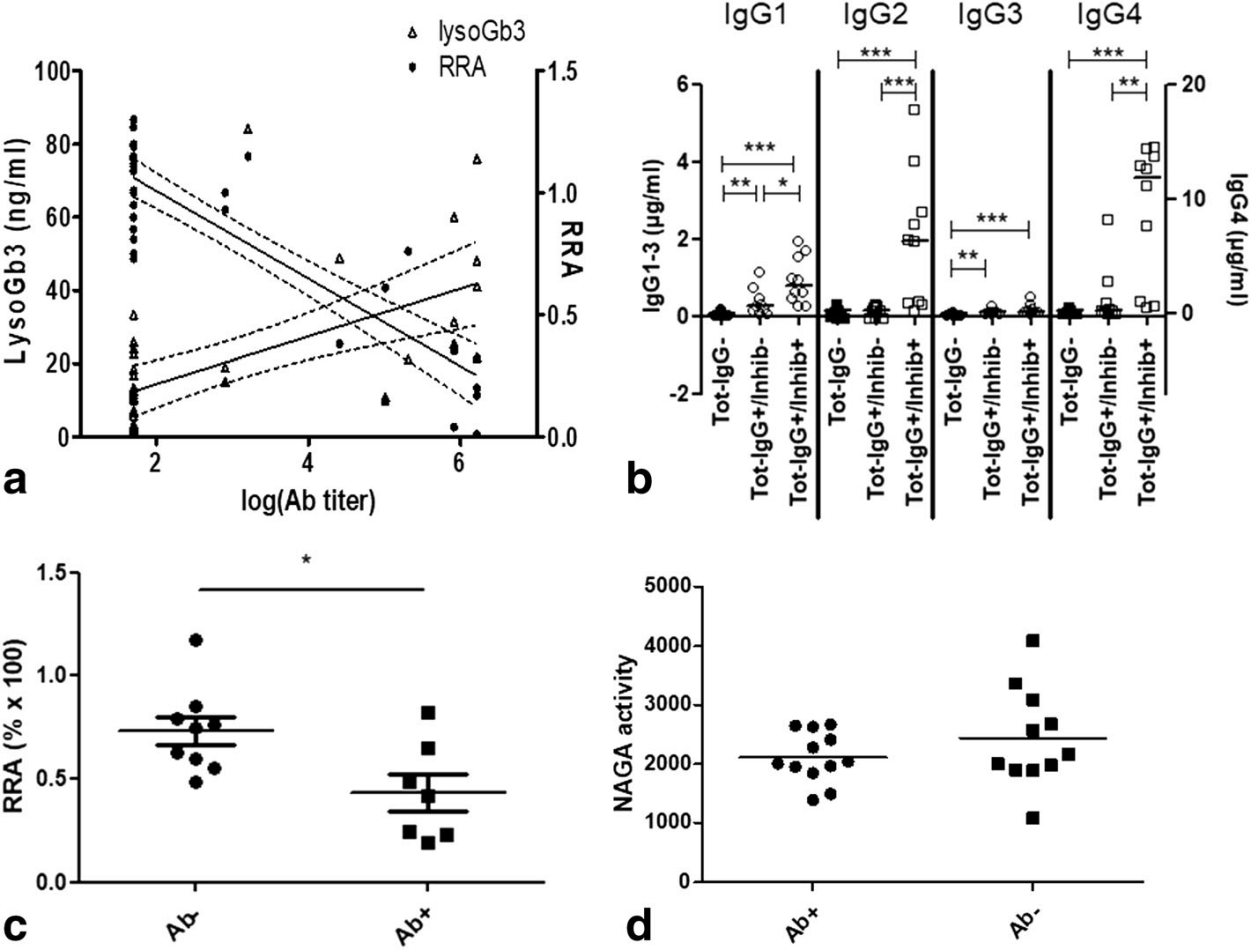
**a** Antibody titers are correlated with the *lyso*Gb3 plasma levels (Spearman *r* = 0.63, *p* < 0.0001) and inversely correlated with the residual enzymatic activity (RRA% × 100) of agalsidase in sera (Spearman *r* = − 0.74, *p* < 0.0001). **b** IgG1–4 subclasses levels (μg/ml) depending on total IgG status (Tot-IgG) and inhibition (inhib+ corresponding to RRA < 0,74), Mann-Whitney test (*: *p* < 0,05; **: *p* < 0,01; ***: *p* < 0,001). **c** Alpha-galactosidase A activity is lower in leucocytes cultured with Ab-positive compared to Ab-negative serum (Mann-Whitney test, median: 42.0 (*n* = 7) vs 75.6 (*n* = 9), *p* = 0.04). **d** Anti-agalsidase antibodies have no effect on alpha-galactosidase B (NAGA): there is no difference in NAGA enzyme activity when incubated with Ab-positive or Ab-negative serum (*n* = 12 and 11 respectively, unit in pmol/min/μg; Mann-Whitney test, *p* = 0.44)

The neutralizing assay in serum was performed in all the men. In the men exposed to agalsidase, the enzyme RRA was correlated with the antibody titer (Spearman’s test, *r* = − 0.67, *p* < 0.0001, Fig. 4a). When considering the Ab-positive serum alone, the RRA was correlated with all subclasses, especially IgG4 (Spearman’s test, *r* = − 0.75, *p* = 0.0005) and IgG2 (Spearman’s test, *r* = − 0.72, *p* = 0.001, Fig. 4b and Table 2). Inhibition was confirmed intracellularly with decreased enzymatic activities in Fabry patient PBMCs cultured with agalsidase when IgG-positive serum (Ab titers 100–1,638,400) was added (Mann-Whitney test, median: 42.0 (*n* = 7) vs 75.6 (*n* = 9), *p* = 0.04, Fig. 4c). Whereas the alpha-galactosidase B also known as alpha-N-acetylgalactosaminidase (NAGA) enzyme shares 46 to 62% homology in its amino acid sequence with alpha-galactosidase A [12], no difference in NAGA activity was found after incubation with either IgG-positive or IgG-negative sera (Mann-Whitney test, *p* = 0.44, Fig. 4d), suggesting the specificity of the inhibition.

In the men currently treated with agalsidase, the correlation between lysoGb3 plasma levels and RRA was almost significant (Spearman’s test, *r* = − 0.34, *p* = 0.056, *n* = 33); the trend remained when focused on the classic phenotype alone (Spearman’s test, *r* = − 0.43, *p* = 0.055, *n* = 21) but not when focused on the non-classic phenotype (Spearman’s test, *r* = − 0.004, *p* = 0.99, *n* = 12). No inhibition (RRA > 74%) was detectable in 7 Ab-positive men and the two Ab-positive women. Although the antibody titers were lower in the inhibition-negative/Ab-positive men (median 1/800 [IQR 1/600–1/103,200], *n* = 7) than in the inhibition-positive/Ab-positive men (1/1,229,000 [1/281,600–1/1,638,000], *n* = 11, Mann-Whitney test, *p* = 0.01), the lysoGb3 plasma levels were not different (Mann-Whitney test, median 20.0 ng/ml [IQR 16.0–68.4] vs 44.5 [24.5–55.6], *n* = 4 and 7 respectively, *p* = 0.26).

When only the inhibition-negative classic patients were considered, there was a trend towards higher *lyso*Gb3 plasma levels in the Ab-positive patients than in the Ab-negative patients from the classic clusters only (Mann-Whitney test, median: 20.0 ng/ml, IQR [16.0–68.4], *n* = 4 vs 11.6 [9.4–19.8], *n* = 10, *p* = 0.076, Fig. 4d) despite the absence of differences in the cumulative exposure to agalsidase (*p* = 0.7).

No inhibition was observed in the women exposed to agalsidase (*n* = 25) including the 2 Ab-positive patients.

## Discussion

This study presents one of the largest independent cohorts of patients screened for anti-agalsidase antibodies. Morerover, we used an anti-agalsidase specific ELISA which is a more sensitive approach than serum inhibition assays that have been used in the very recent literature [13, 14]. LysoGb3 has been implicated in FD pathophysiology and is actually the best biomarker for FD severity [15–17]. Higher lysoGb3 plasma levels have been observed in classic phenotype and in Ab-positive patient separately [13, 17]. Regardless of the agalsidase molecule administered (alfa or beta), we show that antibodies are more likely to develop in men with classic phenotype. In these classic patients, lysoGb3 plasma levels remain higher in Ab-positive patients. This suggests that antibodies are associated with a more severe disease. Interestingly, six of the eight transplanted patients had antibodies, a prevalence that can be underestimated since the patients have immunosuppressants. However, whether the antibodies are directly involved in the disease severity or simply accompany the severe phenotype is unknown.

Antibodies have been suggested to worsen the prognosis by inhibiting the ERT [13, 14]. Indeed we observe a clear correlation between inhibition and antibody titers, nevertheless the correlation between inhibition and lysoGb3 was not significant and inhibition was undetectable in some Ab-positive serum despite high lysoGb3 levels. Hence inhibition-assays alone clearly lack sensitivity for assessing the humoral immune response: we share the findings of Lenders et al. in the higher IgG4 levels associated with inhibition, nevertheless, by using specific anti-agalsidase IgG subclasses ELISA, we can affirm that IgG1, IgG2 and IgG3 are also observed, moreover we did not observe inhibition in some IgG4-positive patients [14]. The polyclonality, reflected by the different existing IgG subclasses, suggests that antibodies can target different epitopes of the agalsidase with multiple and unpredictable consequences [18]. IgG4 that were well correlated with Ab-titers, can bind an epitope such as agalsidase, and result in possible inhibition; however, their physical properties lead this IgG subclass to form bispecific Abs that are functionally monovalent, unable to form large immune complexes and incapable of activating the classical complement pathway [19]. To our knowledge, moreover, no immune complexes or complement deposits have been observed with antibodies in renal biopsies of Fabry patients. Finally, despite benefits from anti-IgG4 antibodies in vitro in rescuing enzyme activity in ERT inhibition positive patients [14], benefits from immunosuppressive drugs have been unobvious in terms of Gb3 clearance and enzyme activity recovering in Fabry mice [20] as well as in patients in vivo: Lenders et al. recently reported that despite a decrease in antibodies under immunosuppressants for renal transplant, the *lyso*Gb3 levels remained stable [21]. The immune response developed towards ERT cannot be limited to a quantitative enzymatic approach.

Another concept links antibodies to clinical phenotype with a qualitative approach: the men with classic phenotypes have the lowest residual enzymatic activities, having therefore higher lysoGb3 levels and being more prompt to develop antibodies. Thus, antibodies would be a hallmark for Fabry disease severity.

The epidemiology of Fabry disease is changing with a higher proportion of non-classic presentations [22]. Severity and prognosis differ according to these clinical phenotypes [23]. There is an urgent need to assess the benefits of ERT according to the clinical phenotypes. Anti-agalsidase antibodies, as a hallmark of severity, could play a role in the stratification of the groups. Because decreasing antibody titers is not sufficient to improve the prognosis in the severe Ab-positive patients [21], other therapeutic approaches should be evaluated, such as increasing the dose of agalsidase [14] or adding chaperone molecule.

The main limitation of this study was the retrospective analysis of clinical data, although the standardized form with automated scoring did limit this bias. Also, antibodies are known to develop within the first six months of ERT [24] and then disappear in some tolerant patients [24, 25]*,* we studied only a single time point and thus could not comment on immunotolerance (either natural or induced by immunosuppressants). Also, we did not perform the neutralizing test in all the women, as we did not observe inhibition in any of the 34 women tested including all the treated patients. Whereas these findings were in accordance with the literature [13], we did not apply the inhibition assay to the rest of the untreated women cohort.

## Conclusion

Anti-agalsidase antibodies almost exclusively develop in men with a severe classic Fabry phenotype and are associated with higher lysoGb3 plasma levels. Despite being frequently inhibitor, anti-agalsidase antibodies have no obvious clinical impact although their association with lysoGb3 levels could be considered as a hallmark of severity associated to the classic phenotype.

## Abbreviations

Ab: Antibody
ANCOVA: Analysis of covariance
BSA: Bovine serum albumin
CRIM: Cross-reactive immunologic material
DPBS: Dulbecco’s phosphate-buffered saline
eGFR: Estimated glomerular filtration rate
ELISA: Enzyme-linked immunosorbent assay
ERT: Enzyme replacement therapy
FD: Fabry disease
Gb3: Globotriaosylceramide
HCM: hypertrophic cardiomyopathy
HR: Hazard ratio
HRP: Horseradish peroxidase
IQR: Interquartile range
LysoGb3: Globotriaosylsphingosine
MDRD: Modification of Diet in Renal Disease
MTPs: Mutations leading to truncated alpha-galactosidase proteins
NAGA: Alpha-N-acetylgalactosaminidase
OR: Odds ratio
PBMCs: Peripheral blood mononuclear cells
RRA: Residual relative activity
UPLC-MS/MS: Ultra-performance liquid chromatography coupled to tandem mass spectrometry
